# Supplementary material for: Cicadomorpha Community (Hemiptera: Auchenorrhyncha) in Portuguese Vineyards with Notes of Potential Vectors of Xylella fastidiosa
Source: Insects. 2023 Mar 2;14(3):251. doi: 10.3390/insects14030251 (PMC10057602; doi:10.3390/insects14030251)
Supplement: Supplementary file 1 [file insects-14-00251-s001.zip › insects-2248560-supplementary.pdf]

Table S1. Vineyards' information: sampling dates (2018 and 2019), metric characteristics, and management data.

Vineyards	2018 Sampling dates			2019 Sampling dates			Y	X	Elevation (m)	Spacing (m)	Variety	Training System	Insecticide	Herbicide	Fungicide	Production mode	Soil management
	LS	S	A	LS	S	A											
V1	27/jun	30/aug	19/oct	16/jul	-	21/oct	40.213508	-8.455542	24,219	2.10 x 0.90	Marselan	Cordon de Royat (unilateral)	Lambda-Cyhalothrin - July/ August	Glyphosate + Oxifluorfen - February	Mancozeb + Metalaxyl M - March/ April; Mancozeb - April; Wettable sulfur - April/ May; Cymoxanil + Folpet-April; Folpet + Fosetil al. + Iprovalicarb - May; Metirame + Piraclostrobin - May/ June; Kresoxim-methyl + diphenoconazole - June; Copper Oxychloride - June; Penconazole - July/ August; Pirimetanil - July/ August	Integrated Production	Tillage between vines
V2	27/jun	30/aug	19/oct	16/jul	-	21/oct	40.207294	-8.451814	43,258	2.30 x 1	Castelão; Baga, Bical, Arinto	Cordon de Royat (bilateral)	Lambda-Cyhalothrin - July/ August	Glyphosate + Oxifluorfen - February	Mancozeb + Metalaxyl M - March/ April; Mancozeb - April; Wettable sulfur - April/ May; Cymoxanil + Folpet-April; Folpet + Fosetil al. + Iprovalicarb - May; Metirame + Piraclostrobin - May/ June; Kresoxim-methyl + diphenoconazole - June; Copper Oxychloride - June; Penconazole - July/ August; Pirimetanil - July/ August	Integrated Production	Tillage between vines
V3	27/jun	30/aug	19/oct	16/jul	24/sep	21/oct	40.472186	-8.55042	46,770	2.5 x 1	Typical varieties of the region	Unilateral Cordon	Lambda-Cyhalothrin - July/ August	Glyphosate + Oxifluorfen - February	Mancozeb + Metalaxyl M - March/ April; Mancozeb - April; Wettable sulfur - April/ May; Cymoxanil + Folpet-April; Folpet + Fosetil al. + Iprovalicarb - May; Metirame + Piraclostrobin - May/ June; Kresoxim-methyl + diphenoconazole - June; Copper Oxychloride - June; Penconazole - July/ August; Pirimetanil - July/ August	Integrated Production	No

V4	27/jun	30/aug	19/oct	16/jul	24/sep	21/oct	40.472386	-8.555479	59,408	2.5 x 2	Typical varieties of the region	Unilateral Cordon	Lambda-Cyhalothrin - July/ August	Glyphosate + Oxifluorfen - February	Mancozeb + Metalaxyl M - March/ April; Mancozeb - April; Wettable sulfur - April/ May; Cymoxanil + Folpet- April; Folpet + Fosetil al. + Iprovalicarb - May; Metirame + Piraclostrobin - May/ June; Kresoxim-methyl + diphenoconazole - June; Copper Oxychloride - June; Penconazole - July/ August; Pirimetanil - July/ August	Integrated Production	No
V5	27/jun	30/aug	19/oct	16/jul	24/sep	21/oct	40.461199	-8.531627	54,375	2.30 x 1	Typical varieties of the region	Unilateral Cordon	Lambda-Cyhalothrin - July/ August	Glyphosate + Oxifluorfen - February	Mancozeb + Metalaxyl M - March/ April; Mancozeb - April; Wettable sulfur - April/ May; Cymoxanil + Folpet- April; Folpet + Fosetil al. + Iprovalicarb - May; Metirame + Piraclostrobin - May/ June; Kresoxim-methyl + diphenoconazole - June; Copper Oxychloride - June; Penconazole - July/ August; Pirimetanil - July/ August	Integrated Production	No
V6	27/jun	30/aug	19/oct	16/jul	-	21/oct	40.212725	-8.454453	27,714	2.30 x 0.90	Touriga Nacional; Tinta Roriz; Alfrocheiro	Cordon de Royat (unilateral)	Lambda-Cyhalothrin - July/ August	Glyphosate + Oxifluorfen - February	Mancozeb + Metalaxyl M - March/ April; Mancozeb - April; Wettable sulfur - April/ May; Cymoxanil + Folpet- April; Folpet + Fosetil al. + Iprovalicarb - May; Metirame + Piraclostrobin - May/ June; Kresoxim-methyl + diphenoconazole - June; Copper Oxychloride - June; Penconazole - July/ August; Pirimetanil - July/ August	Integrated Production	Between vines
V7	-	-	-	19/jun	23/sep	21/oct	40.140458	-7.512300	473,104	3 x 1.50	Touriga Nacional; Trincadeira, Syrah	Bilateral Cordon	-	Glyphosate - March	Mancozeb + cymoxanil - May; Wettable sulfur- June; Fluopyram + tebuconazole - July	Integrated Production	Vegetation cover mowing - June and August
V8	-	-	-	19/jun	23/sep	21/oct	40.164472	-7.520331	439,722	3 x 1	Touriga Nacional, Jaen	Bilateral Cordon	-	-	Wettable sulfur - April	Integrated Production	Vegetation cover mowing - March and June
V9	-	-	-	19/jun	23/sep	21/oct	40.168936	-7.510456	409,972	3 x 1	Jaen, Rufette, Syrah	Bilateral Cordon	-	-	Wettable sulfur - April	Integrated Production	Vegetation cover mowing - April and June

V10	-	-	-	19/jun	23/sep	21/oct	40.326719	-7.418683	459,561	3 x 1	Jaen, Moscatel, Maroco	Bilateral Cordon	-	Glyphosate - April;	Azoxystrobin + Folpet - May; Mancozeb + metalaxyl-M - June; Tetraconazole - June; Fenebuconazole - July	Integrated Production	Vegetation cover mowing - April and June
V11	-	-	-	19/jun	23/sep	21/oct	40.317892	-7.302781	484,359	3 x 2	Touriga Nacional	Unilateral Cordon	-	Glyphosate - April;	Wettable sulfur - April; Dimethomorph + Dithianon - April/June; Penconazole - June	Integrated Production	Vegetation cover mowing - April and June
V12	-	-	-	27/jul	26/sep	25/oct	41.1485948	-7.1271711	151,303	2.20 x 1	Sousão	Cordon de Royat (bilateral)	-	-	Wettable sulfur + Fosetyl aluminium - March; Cymoxanil + Folpet + Fosetyl aluminium + Spiroxamine - April; Kresoxim-methyl and Penconazole -June; Boscalid + Kresoxim-methyl - July	Integrated Production	Vegetation cover mowing - March, April and May
V13	4/jul	29/aug	16/oct	27/jul	26/sep	25/oct	41.184728	-7.109831	133,797	2 x 10	Tinta Cão	Unilateral Cordon	-	-	Wettable sulfur + Fosetyl aluminium - March; Mancozeb -May/ June	Integrated Production	No
V14	11/jul	29/aug	16/oct	27/jul	26/sep	22/oct	41.224727	-7.091073	125,710	2.20 x 0.95	Tourina Nacional	Cordon	-	-	Wettable sulfur + Fosetyl aluminium - March; Mancozeb -May	Integrated Production	No
V15	-	-	-	27/jul	26/sep	24/oct	41.1169499	-7.9869087	211,473	2 x 1.2	Viognier	Cordon de Royat (unilateral)	-	-	Sulfur - April/ May/ June; Copper - April/ May/ June	Organic	Vegetation cover mowing - in March and April
V16	-	-	-	27/jul	26/sep	24/oct	41.1550118	-7.7978446	77,685	2 x 1	Touriga Nacional, Tinta Roriz, Touriga Franca, Tinta Barroca	Cordon de Royat (unilateral)	-	Glyphosate - March;	Sulfur - May; Mandipropamid + zoxamid - May	Integrated Production	No
V17	-	-	-	27/jul	26/sep	24/oct	41.154265	-7.687574	176,179	4.5 x 0.90	Touriga Franca	Cordon de Royat (unilateral)	-	-	Sulfur - April/ May/ June; Copper - April/ May/ June	Organic	Vegetation cover mowing - February and June
V18	-	-	-	27/jul	26/sep	24/oct	41.171111	-7.556944	289,758	2.30 x 0.80	Touriga Nacional	Cordon de Royat (unilateral)	-	Glyphosate - March;	Sulfur - May; Mandipropamid + zoxamid - May	Integrated Production	No
V19	-	-	-	27/jul	26/sep	24/oct	41.175709	-7.530771	267,565	2.5 x 1	Tinta Francista, Touriga Nacional, Vinhas Velhas	Cordon de Royat (unilateral)	-	Glyphosate - March;	Folpet + Metalaxyl - April/ May; Sulfur and Penconazole - April; Fluopyram + tebuconazole - June; Copper sulfate - July; Boscalid + Kresoxim-methyl - July	Integrated Production	No
V20	-	-	-	27/jul	26/sep	24/oct	41.180833	-7.476667	285,160	2.20 x 1	Touriga Nacional, Touriga Franca,	Cordon de Royat (unilateral)	-	Glyphosate - March;	Folpet + Metalaxyl - April/ May; Sulfur and Penconazole - April; Fluopyram + tebuconazole - June; Copper	Integrated Production	No

											Tinta Roriz, Tinto Cão				sulfate - July; Boscalid + Kresoxim-methyl - July		
V21	-	-	-	27/jul	26/sep	24/oct	41.108056	-7.241389	300,447	2 x 1	Touriga Franca	Cordon de Royat (bilateral)	-	-	Folpet + Metalaxyl - April/ May; Sulfur and Penconazole - April; Fluopyram + tebuconazole - June; Copper sulfate - July; Boscalid + Kresoxim-methyl - July	Integrated Production	No
V22	26/jun	11/sep	19/oct	15/jul	23/sep	21/oct	38.522541	-8.953211	50,382	3 x 1	Tinta Roriz, Touriga Nacional, Tinta Amarela	Unilateral Cordon	-	-	Mancozeb + cymoxanil - May; Wettable sulfur- June; Fluopyram + tebuconazole - July	Integrated Production	No
V23	26/jun	11/sep	19/oct	15/jul	23/sep	21/oct	38.567994	-8.928173	99,902	3 x 1	Moscatel Roxo, Touriga Nacional	Unilateral Cordon	-	-	Mancozeb + cymoxanil - May; Wettable sulfur- June; Fluopyram + tebuconazole - July	Integrated Production	No
V24	26/jun	11/sep	19/oct	15/jul	-	-	38.490498	-9.022675	108,713	3 x 1	Fernão Pires, Moscatel de Setúbal	Unilateral Cordon	-	-	Mancozeb + cymoxanil - May; Wettable sulfur- June; Fluopyram + tebuconazole - July	Integrated Production	No
V25	26/jun	11/sep	19/oct	15/jul	23/sep	21/oct	38.540465	-8.985373	96,520	3 x 1	Castelão, Trincadeira e Touriga Nacional	Unilateral Cordon	-	-	Mancozeb + cymoxanil - May; Wettable sulfur- June; Fluopyram + tebuconazole - July	Integrated Production	No
V26	11/jul	29/aug	16/oct	15/jul	17/sep	22/oct	41.5162149	-7.092967	344,346	2 x 1	Touriga-Franca; Sousão	Unilateral Cordon	-	-	Sulfur and Copper - June/ July	Organic	Vegetation cover mowing - June
V27	-	-	-	25/jun	25/sep	22/oct	41.550195	-7.259052	260,920	2 x 1	Touriga Franca, Touriga Nacional, Bastardo,	Unilateral Cordon	-	-	Wettable sulfur - May/ June/ July	Integrated Production	Vegetation cover mowing - March and June
V28	-	-	-	25/jun	25/sep	22/oct	41.59775	-7.363637	510,649	2 x 2	Viosinho, Códega do Larinho, Malvasia Fina,	Unilateral Cordon	-	-	Wettable sulfur - May/ June/ July	Integrated Production	Vegetation cover mowing - March and June
V29	11/jul	29/aug	19/oct	26/jun	25/sep	22/oct	41.6473833	-7.5843222	385,721	2 x 1	Alvarinho	Unilateral Cordon	-	-	Malcozeb + cymoxanil - May; Wettable sulfur- June; Fluopyram + tebuconazole - July;	Integrated Production	Vegetation cover mowing - June
V30	11/jul	29/aug	16/oct	26/jun	1/oct	22/oct	41.292179	-7.112580	393,805	2 x 0.90	Touriga-Franca, Touriga	Cordon	-	-	Wettable sulfur + Fosetyl aluminium - March; Mancozeb -May;	Integrated Production	No

V31	22/jun	28/aug	26/oct	8/jul	25/sep	21/oct	41.680022	-8.53092	168,628	3 x 1	Nacional, Rabigato, Espadeiro, Borraçal, Alvarinho	Unilateral Cordon	Deltamethrin - June/July	-	Folpet + Metalaxyl - April/ May; Mancozeb + cymoxanil - May; Wettable sulfur- June; Fluopyram + tebuconazole - July;	Integrated Production	No
V32	22/jun	28/aug	26/oct	8/jul	24/sep	21/oct	41.678658	-8.531356	165,049	3 x 1	Alvarinho	Unilateral Cordon	Deltamethrin - June/July	-	Folpet + Metalaxyl - April/ May; Mancozeb + cymoxanil - May; Wettable sulfur- June; Fluopyram + tebuconazole - July;	Integrated Production	No
V33	22/jun	28/aug	26/oct	8/jul	25/sep	21/oct	41.785855	-8.494984	68,918	3 x 1	Alvarelhão, Borraçal, Pedral	Unilateral Cordon	Deltamethrin - June/July	-	Folpet + Metalaxyl - April/ May; Mancozeb + cymoxanil - May; Wettable sulfur- June; Fluopyram + tebuconazole - July;	Integrated Production	No
V34	22/jun	28/aug	26/oct	8/jul	25/sep	21/oct	41.815375	-8.410264	57,641	3 x 1	Amaral, Rabo de Anho, Vinhão	Unilateral Cordon	Deltamethrin - June/July	-	Folpet + Metalaxyl - April/ May; Mancozeb + cymoxanil - May; Wettable sulfur- June; Fluopyram + tebuconazole - July;	Integrated Production	No
V35	22/jun	28/aug	26/oct	8/jul	25/sep	21/oct	41.792375	-8.538943	39,478	3 x 1	Vinhão, Espadeiro	Unilateral Cordon	Deltamethrin - June/July	-	Folpet + Metalaxyl - April/ May; Mancozeb + cymoxanil - May; Wettable sulfur- June; Fluopyram + tebuconazole - July;	Integrated Production	No

LS – Late spring; S – Summer; A – Autumn; Y – Latitude; X – Longitude.
